# Menstrual Cycle Hormonal Changes and Energy Substrate Metabolism in Exercising Women: A Perspective

**DOI:** 10.3390/ijerph181910024

**Published:** 2021-09-24

**Authors:** Anthony C. Hackney

**Affiliations:** 1 Department of Exercise & Sport Science, University of North Carolina, Chapel Hill, NC 27599, USA; ach@email.unc.edu; Tel.: +1-919-962-0334; 2 Department of Nutrition, Gillings School of Global Public Health, University of North Carolina, Chapel Hill, NC 27599, USA

**Keywords:** estrogens, progesterone, sports, performance, female, eumenorrhea

## Abstract

This article discusses the research supporting that the hormonal changes across the menstrual cycle phases affect a woman’s physiology during exercise, specifically addressing aspects of energy substrate metabolism and macro-nutrient utilization and oxidation. The overarching aim is to provide a perspective on what are the limitations of earlier research studies that have concluded such hormonal changes *do not affect* energy metabolism. Furthermore, suggestions are made concerning research approaches in future studies to increase the likelihood of providing evidence-based data in support of the perspective that menstrual cycle hormonal changes *do affect* energy metabolism in exercising women.

## 1. Introduction

The number of women engaged in physical exercise for health and sporting endeavors has grown exponentially in recent decades [1]. Coinciding with this occurrence, the number of research studies examining female physiological responses to exercise has grown too. The total amount of female-based research is less than that conducted in men, and far more research is needed [1]. This last point is especially warranted due to the unique characteristics of female physiology, specifically the influence of the fine-tuned interactions of the menstrual cycle, ovarian cycle, and female reproductive hormonal changes found in women. Evidence has mounted in recent years that these unique hormonal changes may preclude the application of many of the physiological findings in male-based studies directly to women [1,2,3].

Physiologically, the female sex steroid hormones (i.e., estrogens, progesterone) have a variety of biological roles other than regulating just reproductive function (i.e., pleiotropic effects) [4,5,6]. In exercising women one of these alternative roles is influencing the substrate metabolism of carbohydrate and fat utilized in ATP energy production [6,7]. To this end, it is well established that the availability and utilization of energy substrates during an exercise session are critically instrumental in enhancing human physical performance capacity [8,9]. This latter point is especially true for carbohydrates since the storage capacity in humans for this macro-nutrient is limited, and it is essential in exercise metabolism, especially at higher intensities of activity [8,9].

The circulating levels of female sex steroid hormones (FSSH) fluctuate in eumenorrheic women during their reproductive years as they progress through the phases of their menstrual cycle (i.e., menses, follicular, ovulation, luteal) [5,10]. As such, these changes in FSSH across the menstrual cycle can potentially mediate aspects of carbohydrate and fat metabolism at rest or in response to an exercise bout at different times in the cycle. Researchers have examined such effects in animals and humans for nearly 50 years. Notedly, while both estrogens and progesterone have non-reproductive functions [11], exercise studies have primarily focused on the aspects of estrogens to influence metabolism (*N.B.,* the term “estrogens” is used herein collectively to refer to estrone, estriol, and estradiol-β-17; the latter being the predominate hormonal form in women [11]). Animal-based evidence clearly supports the impactful actions of estrogens on metabolism, and some, but not all human-based research concurs [4,5,6,11]. To date, due to conflicting findings between human studies, no consensus in the research community exists on the efficacy, uniformity, and magnitude (if any) of the effect of estrogen changes throughout the menstrual cycle phases on substrate metabolism. As a consequence, many scientists in the research community can essentially be divided into two schools of thought:

- Those who believe menstrual cycle hormonal changes *do affect* a woman’s metabolism during exercise, and
- Those who believe the menstrual cycle hormonal changes *do not affect* a woman’s metabolism during exercise.

Accordingly, the intent of this commentary article is to address select aspects of this topic. Especially, it presents those factors that have historically led potentially to the ambiguity in the research findings on this topic and to argue for the perspective that the menstrual cycle hormonal changes *do affect* a woman’s energy metabolism during exercise.

Organizationally this article presents, (a) background evidence of the *do affect* research studies supporting menstrual cycle hormonal changes on energy substrate metabolism, (b) factors in prior *do not affect* studies confounding and limiting their findings, and (c) commentary (recommendations) throughout for improving research in this area. For those readers unfamiliar with the physiology and endocrinology of the menstrual cycle, select review articles [5,10] are recommended for an overview of these topics.

## 2. Research Background

### 2.1. Muscle Glycogen—Glucose

Most studies in women have not detected any difference in energy substrate metabolism at rest between menstrual cycle phases [12], but stored energy reserves are reported to differ. Especially, muscle glycogen content (*vastus lateralis*) at rest is higher in the luteal phase (LP, high FSSH levels) than in the follicular phase (FP, low FSSH levels) of the menstrual cycle when dietary carbohydrate intake is standardized [13]. This was speculated to be an LP induced glycogen sparing effect brought on via an enhanced reliance on fat as an energy source. However, other researchers noted that resting (free-living) food selection in physically active women shifts towards greater levels of carbohydrate consumption in the LP, thereby perhaps leading to increases in glycogen synthesis and storage [14]. In agreement with this last point, Becket et al. found estrogens increase muscle glycogen synthase activity in rodent-model research [15].

A number of studies report submaximal steady-state exercise when performed in the LP versus FP results in differences in muscle glycogen utilization [16,17,18]. For example, during a standardized 60-min cycling bout (~70% maximal oxygen uptake [VO_2max_] in both the LP and FP), a muscle glycogen sparing effect was observed in the LP [17]. Again, this is presumed to be due to increasing fat utilization occurring and as such inducing the glycogen sparing during the LP. Supportively, Zderic et al. found the rate of appearance (R*a*; from hepatic glycogenolysis) and disappearance (R*d*) of glucose were lower in LP than FP during cycling exercise (at ~90% lactate threshold) [19]. Similarly, Devries et al. reported glucose R*a* and R*d* and total glycogen utilization were lower in LP than FP during endurance exercise (90 min cycling at 65% of VO_2max_) [18]. These authors also attributed their findings to the higher concentration of estrogens in the LP promoting increased muscle glycogen storage (rest) and sparing during exercise [18,20]. Interestingly, the influences of the estrogens to affect hepatic glucose output has been detected only at exercise intensities that are sufficiently elevated to increase the glucose utilization demand above a certain level [19,21].

### 2.2. Substrate Oxidation

Hackney et al. found higher circulating estrogens levels (either via endogenous (menstrual phase) or exogenous (pharmacological estrogens administration)) resulted in higher fat oxidation and lower carbohydrate oxidation during endurance running (60 min at 65% of VO_2max_) compared with lower estrogens conditions [22]. Similarly, Zderic et al. found the carbohydrate oxidation rates were lower and fat oxidation rates higher in LP than in FP during cycling exercise (at ~90% of the lactate threshold) [19]. These findings were supported by several other studies [7,23,24,25,26]. Furthermore, Wenz et al. found that the greater fat oxidation in LP exercise is magnified by a period of low carbohydrate dietary intake [26]. Additionally, the variance in substrate oxidation between the LP and FP seems intensity-dependent, and as exercise exceeds the lactate threshold the demand for increased carbohydrate consumption outstrips the influence of estrogen [7]. While most investigators in these studies attribute the shift toward reduced carbohydrate oxidation, Lariviere et al. noted increased oxidation of the amino acid leucine during LP, could be an additional mechanism for carbohydrate sparing [27].

### 2.3. Lactate

For decades, lactate has been viewed as a mainstay of assessing anaerobiosis in an exercising individual. Forty years ago, Jurkowski and associates first demonstrated that peak lactate response to a standardized exercise session was lower in the LP versus FP [28]. The authors attributed this to the *Ra* of lactate and carbohydrate substrate turnover being less in LP due to changing FSSH concentrations. These results were confirmed in later studies by McCracken et al. [24] and Berend et al. [25] involving intensive/exhaustive exercise. Furthermore, others have reported comparable findings for submaximal exercise levels occurring near the lactate threshold [26,29,30].

### 2.4. Mechanism of Estrogens Action

**Figure 1:**
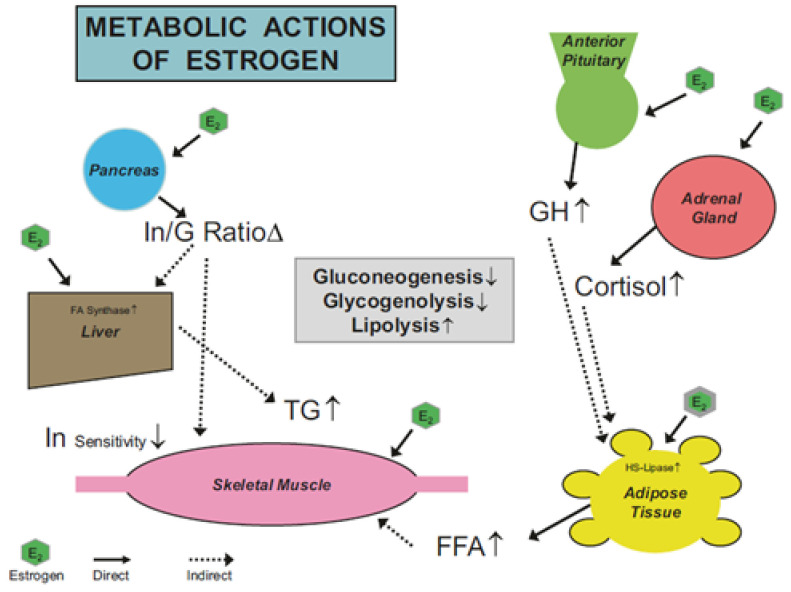
The figure illustrates the direct (^____^) and indirect (----) effects of estrogens (E_2_) on a variety of hormones and physiological processes important to exercise energy metabolism; e.g., energy substrate availability-mobilization, Abbreviations: FFA = free fatty acids, G = glucagon, GH = growth hormones, I insulin, TG = triglycerides. Symbols: ↑ = increase, ↓ = decrease, Δ = change. Taken from [5], used with permission.

The physiological mechanisms for estrogens to influence substrate metabolism are attributed to both direct and indirect actions of the hormone. The direct actions comprise those influences on the lipolytic enzymes involved with the regulation of fatty acid mobilization at the adipose and muscular tissues leading to greater fat than carbohydrate utilization [4,31]. For example, in humans estrogens promote (1) increased lipoprotein lipase and hormone-sensitive lipase activity, (2) increase catecholamine-induced lipolysis, and (3) down regulation of lipogenic-related genes [31]. The indirect actions are those ascribed to the role estrogens play in facilitating other hormones which promote lipolysis as well as glycogenesis (at rest) and mitigating glycogenolysis during exercise (see Figure 1) [4,5,6]. Interestingly, during pregnancy when extremely large increases in FSSH occur, the impact on these indirect hormonal factors is magnified at rest as well as in response to exercise [32]. Regrettably, this latter topic is not well studied.

As noted, both estrogens and progesterone are reported to alter metabolic responses. However, evidence supports the observation progesterone exhibits a largely anti-estrogenic effect [33,34,35]. To this point, D’Eon et al. proposed that a metabolic response to changes in the FSSH occurs only when the estrogens to progesterone ratio is adequately elevated and the magnitude of the increase in estrogen from the FP to LP is in the order of 2-fold or more [36]. However, more research is necessary to assess this interesting supposition before it can be completely accepted or refuted.

## 3. Factors Affecting Research Studies

The prior reviewed evidence supports the *do affect* scientific group, but there is as noted a *do not affect* school of thought group, who propose that the research evidence to support the hormonal changes of the menstrual cycle are inconsequential and demonstrate no menstrual cycle effects on metabolism. In looking back over the last 50 years of research, it is now evident that a number of these such studies have limitations in their approach to the research that calls into question the interpretation of their findings. What follows is my perspective and opinion on some of the most notable limitations found in some of these studies, calling into question the interpretation of these *do not affect* studies and school of thought.

### 3.1. All Women Are Not Alike and as Such, Menstrual Cycles Are Not Alike

Historically, many researchers have assumed that the menstrual cycle in eumenorrheic women is approximately 28 days long, and always shows the classic FSSH changes (i.e., model textbook patterns of responses). As such, if one knows the first day of menstruation, then using a simple forward counting method, and assumes there is ovulation, and that the main phases of the cycle (FP, LP) are equally distributed before and after ovulation, then the cycle phases and hormonal changes can be known approximately. This approach was used for many years in early menstrual cycle research studies. The problem here lies with the assumptions made, which can be totally wrong as evidence shows, for example:

- Cycle length varies *between* women (cycles can be 21 to 35 days, with 2 to 7 days of menses).
- Cycle length can vary *within* a woman by up to 8 days.
- Cycle phase length can also vary *within* women (FP = ±6 days, LP = ±4 days).
- Eumenorrheic women may not always ovulate (e.g., annually with 10–13 cycles a year, a healthy woman could still see one anovulation during this period of time, and as such not see hormonal changes) [37,38,39].

### 3.2. What’s in A Name?—Menstrual Cycle Terminology Confusion

Elliott-Sale and associates in their recent consensus article denoted that one of the problems with past (and some present) menstrual cycle research studies was the lack of clear, definitive terminology on menstrual cycle status and precise operational definitions of cycle phases [10]. This last point is extremely relative; i.e., causing difficulty in comparing study outcomes concerning metabolism (e.g., trying to compare the early FP with the late FP when FSSH values differ greatly is not valid). Furthermore, because of the variability in cycle phase length (discussed prior) some studies have not clearly and specifically indicated the exact point in a menstrual phase when data are collected, creating ambiguous time points for comparisons.

### 3.3. Inadequate Screening for Medical Conditions

Women who appear to be healthy and eumenorrheic may not actually be so. Simply asking if a woman has had her “period” on a regular basis is inadequate screening, but this limited approach to screening has been utilized in past studies [11,37]. Women with polycystic ovary syndrome (PCOS) are an example of how something major can be missed without appropriate medical screening. The prevalence of PCOS is ~6–9% of adult women, and the condition can involve infrequent or prolonged menstrual cycles; but, some women with PCOS have regular monthly cycles [40]. These PCOS women, however, also typically have abnormal FSSH levels which affect their physiological responses to exercise. *N.B*., readers unfamiliar with the physiology and endocrinology of PCOS and related medical conditions affecting FSSH are directed to the review article of Liepa et al. [41] for a synopsis.

### 3.4. A Hormone Is a Hormone, but Biological Specimens Differ

Hormones can be measured in many bodily fluids such as blood, saliva, sweat, urine, and cerebral spinal fluid. Historically, blood has been considered the reference biological specimen that serves as the gold standard. The cost of blood analysis, as well as participant needle phobia, has hampered past research studies, resulting in infrequent hormonal assessments (*N.B*., research on female exercise physiology has been grossly underfunded). Since FSSH levels can vary dramatically across the menstrual cycle, infrequent specimen assessment can mean a researcher may miss the optimal time in a menstrual cycle phase for testing (i.e., the window of peak hormonal change and receptor expression impacting on “down-stream” physiological events (e.g., translation, protein expression, etc.)) [11,42]. Saliva assessment is viewed as the best non-invasive means to overcome the needle-phobia issue and allow more frequent hormonal assessment. Regrettably, saliva hormone level and blood hormone levels do not always perfectly align [42,43,44], for several reasons:

- Saliva is good for assessing free levels of FSSH, while in blood you can examine free, bound, and total levels of such hormones.
- Saliva and blood hormone levels are not always in perfect equilibrium and therefore do not always reflect each other (i.e., efflux from the blood to the saliva can be impeded).
- Not all hormones can be measured in saliva (i.e., molecular weight prevents transport into the saliva).
- Contamination of saliva specimens can be a problem (e.g., blood from brushing teeth, food particles).
- Reference standards for saliva hormone levels are not well established yet.
- Historically early salivary hormonal assays used in prior research studies were problematic, lacking good sensitivity and accuracy.

### 3.5. Statistical Miss-Steps

It is well accepted that research studies require statistical analysis to support the probability of whether the findings are due to experimental design approaches, and are employed to test hypotheses. Statistical analysis is also an accepted tenet and requirement for the publication of most scientific articles. In many exercise studies, the design approach has often been to look at and report group responses, and test those for statistical significance. A problem with such an approach in hormonal work is that studies may have participants who are responders and some who are non-responders (i.e., hormone changes vs. no hormonal changes (i.e., or changes are less than the detection sensitivity of the bioanalytical procedure) [42,45]. Lumping these individuals together dilutes the potential effects of examining the hormonal changes on the physiological outcome measurements (e.g., metabolism). Too many studies (past and present), in attempting to have adequate sample sizes, have kept non-responders in their study sample in an attempt to have adequate statistical power (perhaps being entirely unaware of the fact they have subjects who could be considered non-responders). In lieu of group responses, individual responses should be assessed, and the removal of non-responders considered. Some would consider this “cherry-picking” of the data, but the underlying premise of studying the influence of menstrual cycle hormonal changes is simply that *such changes are necessary* if a physiological response is going to be evoked.

## 4. Discussion

Obviously, the points raised herein are a one-sided perspective. It is important to state that not all studies which fall into the *do not affect* category (school of thought) are poor scientific research. To take that stand would be a gross misrepresentation of one of the tenets of the Scientific Method (i.e., not being open to alternative opinions). That said, over the years there have been a substantial number of these *do not affect* studies that have not carefully considered the nuances of female physiology in their menstrual cycle and hormonal research. Yes, these are peer-review published research, but that does not make them without limitations (i.e., scientific weaknesses in approach, execution, or interpretation). As such, these limitations have resulted in part to the ambiguous nature of the current body of literature on exercise and the menstrual cycle. The *do affect* studies cited herein are also not without limitations either. For example, many of the substrate oxidation studies previously reviewed used respiratory gases to assess carbohydrate and fat contributions to exercise metabolism, which was questioned by some [46]; although the method has been validated in this context [47].

Perhaps most importantly, the scientific community and the public at large have finally started to “listen to women” who are exercisers and athletes and take seriously their reports that their training and competition performance are affected by the phases of their menstrual cycle and the resulting hormonal changes [48]. It seems remarkable that it has taken so long for this to occur and scientists are no longer being tone-deaf to the issue.

As just noted, women athletes feel their competitive performance is affected by their menstrual cycle phase. This commentary article does not address performance per se, but this topic is a logical off-shoot of exercise energy substrate metabolism. McNulty and associates conducted an extensive and thorough review of the available literature on this topic and concluded the effects on performance were trivial, but perhaps could nonetheless be important for the elite female athlete [49]. I do not refute their conclusions, but some of the limitations noted herein as well as those reported by Elliott-Sale et al. on the methodology utilized in this research area indicate that past poor scientific approaches in studies hamper how robust the conclusions can be on the performance topic [10].

Finally, in way of summation, it is clear that evidence-based data exist that show the energy substrate metabolism of exercising women is affected by their menstrual cycle phase and the associated FSSH hormonal changes. Elevations in estrogens, in particular, across the cycle seem to promote carbohydrate sparing and increased fat utilization as an energy source. The complete consequences of this occurrence remain to be seen, but certainly have implications for the training and dietary practices of exercising women, especially competitive athletes (see [50] for dietary discussion). Such findings are not universal to all women and research by some refute the influence of the menstrual cycle phase on female exercise physiology all together, however, poor application of the Scientific Method calls one to question these counter perspectives.

## 5. Conclusions

In conclusion, further study is needed on the role of the menstrual cycle on the exercise physiology of women. There is an important need to ensure this research is of the highest quality and standard, and not to continually repeat the mistakes of the past [10]. To this end, it is important to develop a new generation of scientists who will pursue this line of work robustly. Furthermore, in my opinion, we need more of these scientists to be women as they are vital stakeholders who have been in the minority of research ranks for far too long.

## Data Availability

Not applicable.
